# The Impact of a Clinical Asthma Pathway on Resident Education

**DOI:** 10.1155/2018/5472876

**Published:** 2018-03-29

**Authors:** Hina J. Talib, Yonit Lax, Marina Reznik

**Affiliations:** ^1^Division of Adolescent Medicine, Children's Hospital at Montefiore/Albert Einstein College of Medicine, Bronx, NY, USA; ^2^Division of Academic General Pediatrics, Children's Hospital at Montefiore/Albert Einstein College of Medicine, Bronx, NY, USA

## Abstract

Clinical pathways for asthma management decrease hospital cost and length of stay; however little is known about the educational impact of pathways on residents. Pediatric residents at a children's hospital (*N* = 114) were invited to complete a 22-item computerized, anonymous survey 6 months before and 6 months after asthma pathway implementation. The survey assessed pathway use and residents (1) pathway knowledge, (2) attitudes and experiences with managing asthma, and (3) perceived educational benefits. Mean pathway knowledge score increased from the case before to the case after implementation [1.5 ± 1.0 versus 2.6 ± 1.3, *p* < 0.001], as did high preparedness to manage asthma [61% versus 91%, *p* < 0.001] and electronic order set use [28% versus 80%, *p* < 0.001]. The top three educational benefits of the pathway endorsed by residents were application of evidence-based medicine (57%), ability to assess exacerbations (52%), and skill at communicating respiratory status (47%). After implementation, residents' knowledge and preparedness to manage asthma improved as well as many endorsed educational benefits.

## 1. Introduction

Asthma is a chronic respiratory condition that affects more than six million children and adolescents in the United States and is one of the most common causes of childhood disability [1]. Acute, severe asthma is a leading cause of hospitalization in US children, second only to pneumonia [2]. Children in New York City (NYC) suffer rates higher than national ones of asthma-related morbidity and Bronx county, a borough of NYC, where this study takes place, has the highest rates of asthma prevalence, asthma-related hospitalizations, and deaths in NYC [3]. Accordingly, diagnosis and management of children with asthma-related hospitalizations is a core topic in pediatric residency education, yet adherence to clinical guidelines in asthma management is known to be challenging among pediatricians and family medicine physicians [4–8].

In response to the high numbers of children with asthma-related hospitalizations and as recommended by national guidelines, many children's hospitals have developed clinical pathways for asthma management [9]. Clinical pathways are defined as “systematic approaches to guide healthcare professionals in managing a specific problem” [6]. A recent systematic review of seven studies on clinical asthma pathways in hospitals found that pediatric asthma pathways are associated with a decrease in length of stay [10]. However, to our knowledge, their impact on resident education and asthma management skills have not been previously studied.

Clinical pathways guide clinicians towards practicing the most up-to-date evidence-based medicine informed by national guidelines. Barriers to adherence to clinical guidelines may be related to lack of awareness, familiarity, and institutional policies as well as inertia and resistance to change [6]. Similar to pediatricians and family medicine physicians, pediatric residents may face barriers adhering to clinical guidelines and implementation of clinical pathways may help adherence.

A main criticism of clinical pathways used in children's hospitals is that they reinforce “cookbook medicine,” making physicians into technicians by limiting critical thinking, raising concerns that trainees may have negative reactions [8]. On the other hand, clinical pathways may enhance resident education by presenting evidence-based management options. In fact, little is known about the impact of clinical asthma pathway implementation on pediatric resident education and resident experiences as front-line users of new pathway tools and management algorithms. This study aimed (1) to compare resident knowledge, attitudes, and comfort with pediatric asthma management before and six months after implementation of a new inpatient asthma management pathway and (2) to explore resident's perceived educational benefits and barriers with asthma pathway use.

## 2. Materials and Methods

With permission from the residency training program director, we conducted a prospective survey study of pediatric residents' knowledge, attitudes, and satisfaction with an inpatient clinical asthma management pathway before and after implementation at the Children's Hospital at Montefiore (CHAM), located in Bronx county, NY. This is a low income, urban county with a population of 1.4 million and with rates of asthma-related hospitalizations and deaths about five times and three times higher, respectively, than the national average [3]. CHAM admits an estimated 100 children with acute asthma monthly and acute, severe asthma is the hospital's top admitting diagnosis. The pediatric residency program at CHAM is a large academic program with 29 new interns each year.

An interdisciplinary committee, with physician expertise in Adolescent Medicine, Pediatric Hospital Medicine, Pediatric Pulmonary Medicine, Emergency Medicine, and Pediatric Critical Care and representation from nursing, pharmacy, respiratory therapy, and pediatric chief residents, was convened in 2014 to create the CHAM inpatient asthma pathway and associated care tools including a respiratory scoring system, an asthma action plan, and a patient education video. Implementation also included an updated electronic asthma order set (a tool within our electronic medical ordering system that allows for more rapid ordering from a preselected list of orders including medication orders). This care bundle reflected updated clinical guidelines on asthma management. Three months prior to pathway implementation in 2014, residents attended an educational conference introducing the pathway, explaining the development of the pathway, patient inclusion and exclusion criteria, the use of a respiratory score, and the updated evidence-based management recommendations. Those residents who were unable to attend the seminar were given an introductory written presentation for which they were required to affirm their review. Two additional educational seminars reinforcing the pathway were conducted one month before and after implementation. Materials outlining the pathway and associated care tools were disseminated electronically, through printed posters and cards, as well as through a resource link in the electronic ordering system. The survey was administered 6 months before and 6 months after implementation of the pathway using Survey Monkey software (https://SurveyMonkey.com, LLC, Palo Alto CA) in 2014-2015. Trainees were assured that their responses were anonymous and would not impact performance evaluations. As an incentive, respondents were eligible to win one of three $50 Amazon.com gift cards using the software's raffle feature. The study was approved by the Einstein/Montefiore Institutional Review Board.

The 22-item survey included questions identifying training level and experiences with implementation and use of the clinical asthma pathway and tools. The development of the survey was informed by Kirkpatrick's evaluation hierarchy theory used to evaluate educational interventions on multiple levels including “reaction,” “learning,” and “behavior” [11]. In keeping with this theoretical approach, we assessed “reaction” by exploring pathway satisfaction and attitudes, “learning” by a knowledge scale, and “behavior” by pathway and associated clinical care tools use. The survey assessed respondent (1) asthma management knowledge using clinical case scenarios developed for the study reflecting management per the pathway by asking respondents to assign a respiratory score, pick management steps based on respiratory score, select the pathway standard dose of albuterol, identify pathway exclusion criteria, and determine discharge readiness (score: 0–8 correct responses); (2) attitudes and experiences with managing asthma using a 5-point Likert-type scale (1, strongly disagree, to 5, strongly agree) and preparedness to manage asthma (variable dichotomized as low (1-2) versus high (3–5) agreement with feeling prepared); (3) perceived educational benefits and barriers to pathway use utilizing check-all-that-apply type questions. Chi-square and Student's *t*-test were used for analysis where appropriate.

## 3. Results and Discussion

Response rate of pediatric residents was 68/114 (60%) for the presurvey and 60/114 (53%) for the postsurvey, with no differences by postgraduate training year. Of the postimplementation respondents, 32/60 (53%) reported having attended an educational seminar introducing the pathway. All of the residents who did not attend a seminar affirmed their review of the content of the seminar that was electronically distributed to them.

**Figure 1:**
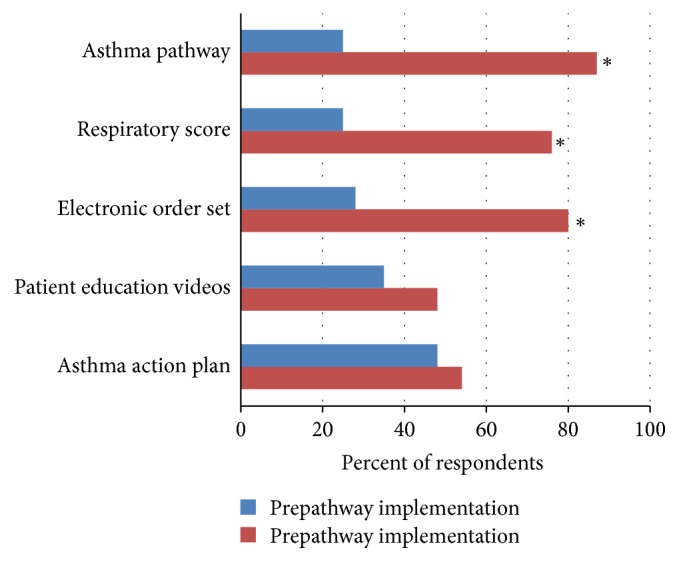
*Resident use of asthma pathway and support tools*. Respondents were asked if they used each of these tools 6 months before and 6 months after asthma management pathway was implemented. Asterisk (*∗*) denotes a significant difference with *p* < 0.001.

Pediatric residents' experiences with asthma management using the pathway and support tools are shown in Figure 1. Overall, 75% of residents had no prior asthma pathway experience and the same percentage, 75%, reported no prior respiratory score use in medical school. As shown, pathway use increased as did use of the existing but updated electronic asthma order set (a tool within our electronic medical ordering system that allows for more rapid ordering from a preselected list of orders including medication orders), while use of asthma action plans and patient education videos did not change, despite these tools being introduced at the educational sessions introducing the pathway.

Mean pathway knowledge score (maximum score = 8 correct) was low but increased from the case before to the case after asthma pathway implementation [1.5 ± 1.0 versus 2.6 ± 1.3, *p* < 0.001]. The highest prescore was 4 and the highest postscore was 6. Pathway scores were not higher in those respondents who attended an in-person educational seminar introducing the pathway. Importantly, more residents reported feeling well prepared to manage acute asthma presentations without supervision (91% versus 60%, *p* < 0.001) after pathway implementation. Overall, after implementation, 54% of the respondents reported satisfaction with the management pathway.

Respondents' attitudes about the pathway's influence on resident education were generally positive both before and after pathway implementation. However, more respondents agreed that pathways did not hinder resident education after implementation compared with the case before implementation (91% versus 76%, *p* < 0.05). We found no difference before and after implementation in respondents' reported likelihood of practicing evidence-based medicine or their agreement that pathways hinder resident autonomy.

The top 3 educational benefits of the pathway endorsed by residents were improved: application of evidence-based medicine (57%); ability to clinically assess exacerbations (52%); and skill at communicating respiratory status (47%). The top 5 barriers to pathway use were disagreement with the content of pathway management (54%); forgetting to use it (50%); lack of education of ancillary staff (50%); lack of access to pathway for reference (24%); and lack of education on pathway use (24%). There was no association between those respondents who cited a lack of education on the pathway use and those who attended an in-person educational seminar introducing the pathway.

This study is the first study, to our knowledge, to explore the effects of implementing a clinical asthma management pathway on pediatric resident education and comfort with asthma management. We found that after pathway implementation, residents' knowledge and comfort managing asthma improved, while their overall attitudes towards clinical pathways remained positive. This contradicts previously published opinions highlighting concerns that clinical pathway management tools hinder resident education and provides evidence for using such tools to enhance pediatric education [6].

In keeping with Kirkpatrick's evaluation hierarchy theory, our study has taken a multilevel approach in evaluating pathway implementation as an educational training tool, with a positive reaction as shown by pathway satisfaction, an educational benefit as demonstrated by the increase in knowledge scale as informed by the pathway, and, finally, an impact on changing behavior through the reported use of the pathway and associated care tools [11]. Based on previous studies, clinical pathways are often implemented with the primary goals of decreasing hospital length of stay and cost [12, 13]. In this paper we provide evidence to suggest an additional goal and benefit of implementing a clinical asthma pathway is its favorable impact on resident education.

A study of pediatric residents conducted about 10 years prior to this study at an academic ambulatory care center in Bronx county demonstrated low competency among pediatric residents in classifying asthma severity, which is the first step towards evidence-based asthma management [14]. Mastering skills to independently manage asthma in both inpatient and outpatient settings is an important focus for every pediatric residency training program. We found in this study that our pediatric residents used and were satisfied with the newly implemented asthma clinical pathway. Our findings suggest that clinical pathways may be considered as a training element during residency for topics like asthma management.

Training in a large urban children's hospital exposes residents to a high-volume learning environment. Implementing a clinical pathway in this environment allows for up-to-date evidence-based practice that may improve future patient care as residents complete their training and graduate to practice in both inpatient and outpatient settings. Notably, our trainees' knowledge of inpatient asthma management as reflected by their low mean knowledge scores was suboptimal prior to the asthma management pathway implementation, with a significant improvement after implementation. Despite improvement, the mean scores were still low 6 months after implementation, suggesting that adoption and integration of the new clinical pathway into knowledge and practice may take longer than 6 months.

Implementation of the clinical asthma management pathway was successful, with high pathway use reported by residents 6 months after implementation. However, there were notable barriers to pathway use. We were surprised to learn that the number one barrier to pathway use (endorsed by more than half of respondents) was disagreement with pathway management and that one-quarter of respondents reported lack of education on pathway use as a barrier. We hypothesize that our pediatric resident respondents experienced discomfort in changing practice in areas where the pathway introduced updated practice management steps. For example, the pathway increased the standard dose of albuterol from 2.5 mg (nebulized) to 5 mg (nebulized), which was a new change from current practice at the institution. Also new to the current standard practice was the fact that the pathway also encouraged the use of metered dose inhalers in hospital settings and gave dosing suggestions for this as well alongside the nebulized doses as an alternative. This highlights an increased need for evidence-based management and further supports the importance of ongoing education of pediatric residents using the literature that provided the evidence for the changes in management.

This study has several limitations. The study was conducted at a single children's hospital. However, our pediatric residency program is similar to those at other large urban, academic children's hospitals in cities with high asthma prevalence rates. In addition, the postimplementation survey was completed 6 months after implementation, and it may take longer to fully institutionalize a new clinical pathway. Lastly, it is inherent in residency training programs that over time a trainee gains knowledge and comfort in managing asthma, and our study was unable to control for this factor. To best address this, we specifically assessed knowledge by asking questions informed by the pathway and that highlighted changes in clinical practice rather than general knowledge about asthma classification and management.

## 4. Conclusions

The findings of this study highlight the educational benefits of a new clinical asthma management pathway for pediatric residents at an urban, academic children's hospital serving a community with rates higher than national asthma prevalence and hospitalization ones. We found that the majority of pediatric residents report using the pathway and they were satisfied with the pathway. Importantly, trainees' comfort and knowledge in managing asthma using management steps that are informed by updated clinical guidelines were improved, which is an educational goal in residency training programs. Attitudes towards clinical pathway use were high before and after implementation, encouraging further exploration on expanding the use of clinical pathways in academic training institutions.

## Appendix

### A. Baseline Survey

*Demographics and Background*

*Please Enter Your 4-Character ID.* Remember to save this ID for follow up surveys – We suggest entering into your phone or personal email under Asthma Pathway Study.

1. What year are you currently in your training?
  (A) Resident - Post-Graduate Year 1
  (B) Resident - Post-Graduate Year 2
  (C) Resident - Post-Graduate Year 3
  (D) Medical Student 4th year – matched into CHAM Pediatrics Residency for the 2014-2015 Academic year
2. When caring for pediatric-age patients, have you used an inpatient asthma pathway for asthma management in medical school or prior to your Pediatrics residency at CHAM?
  - Yes
  - No
3. When caring for pediatric age patients, have you ever used an inpatient Respiratory Score for asthma management in medical school or prior to your Pediatrics residency at CHAM?
  - Yes
  - No
4. How many 4-week inpatient rotations have you had at CHAM?
  - 0
  - 1-2
  - 3-4
  - 5-6
  - >7
5. How many rotations of at least 2 weeks duration at CHAM have you had in the Pediatric Emergency Room?
  - 0
  - 1
  - 2
  - 3
  - 4 or more

*Attitudes on Asthma Education. *How much do you agree or disagree with the following statements:

(6) I believe that clinical pathways or protocols that outline management hinder resident and autonomy
  - Strongly disagree
  - Disagree
  - Neither agree nor disagree
  - Agree
  - Strongly agree
(7) I believe that clinical pathways or protocols that outline management hinder resident and student education
  - Strongly disagree
  - Disagree
  - Neither agree nor disagree
  - Agree
  - Strongly agree
(8) Clinical pathways or protocols enhance resident or student's likelihood to practice evidence-based medicine
  - Strongly disagree
  - Disagree
  - Neither agree nor disagree
  - Agree
  - Strongly agree
(9) I feel well prepared to manage an acute asthma exacerbation in emergency room and inpatient settings* without supervision*.
  - Strongly disagree
  - Disagree
  - Neither agree nor disagree
  - Agree
  - Strongly agree
(10) I believe that the implementation of a CHAM asthma pathway will have a positive effect on my education in residency on asthma management
  - Strongly disagree
  - Disagree
  - Neither agree nor disagree
  - Agree
  - Strongly agree

*Asthma Knowledge and Management*

(11) Case 1: you are in the Pediatric Emergency Room and a 3-year-old girl with a history of bronchiolitis presents to the asthma area. On initial assessment you count her respiratory rate to be 32 and note both subcostal retractions and nasal flaring. On inquiring about her activity level parents report a good appetite but increased cough after playing. On auscultation you hear expiratory wheezing through the entire expiratory phase. What is your next step in management?
  (Select one answer)
  - Give Albuterol once and reassess in 30 minutes
  - Give Albuterol and Ipratropium (combo neb) × 1 and reassess
  - Give Albuterol and Ipratropium (combo neb) × 3
  - Give Albuterol and Ipratropium (combo neb) × 3 and Magnesium sulfate
  - Discharge home with Albuterol and Prednisone prescriptions
(12) Case 2: you are in the Pediatric Emergency Room and a 15-year-old boy with a history of mild intermittent asthma presents to the asthma area. On initial assessment you count his respiratory rate to be 34, and note intercostal retractions. On auscultation he has both inspiratory and expiratory wheezing and is able to count to 7 when asked to count to ten in 1 breath. What is your next step in management?
  (Select one answer)
  - Give Albuterol once and reassess in 30 minutes
  - Give Albuterol and Ipratropium (combo neb) × 1 and reassess
  - Give Albuterol and Ipratropium (combo neb) × 3
  - Give Albuterol and Ipratropium (combo neb) × 3 and Magnesium sulfate
  - Discharge home with Albuterol and Prednisone prescriptions
(13) Case 3: you are in the Pediatric Emergency Room, it is the end of your shift and the 13-year-old girl you had seen in the asthma room just finished her third Albuterol/Ipratropium combo neb. She also received one dose of Prednisone 60 mg by mouth 1 hour ago. On reassessment you find her respiratory rate to be 23, with end expiratory wheezing, and subcostal retractions. On asking her to count to 10 in one breath she is able to complete the task. What is your next step in management?
  (Select one answer)
  - Admit to the hospital for continued asthma management
  - Give Albuterol once and reassess in 30 minutes
  - Give Magnesium Sulfate
  - Discharge home with Albuterol and Prednisone prescriptions
  - Discharge home with Albuterol and Ipratropium
(14) Case 4: you are in the Pediatric Emergency Room, it is the beginning of your shift and you just got sign out about a 5-year-old patient in the asthma room who received prednisone and Albuterol/Ipratropium combo nebs 3 times, and was placed on 40% FiO2 via facemask for oxygen saturation of 87%. On your initial assessment she has an O2 saturation of 91% on 40% facemask, inspiratory and expiratory wheezes, subcostal retractions, and a respiratory rate of 31. Parents note a persistently decreased appetite but state that she has been drinking apple juice while in the ED, and had an increased cough when playing. What is your next step in management?
  (Select one answer)
  - Discharge home with Albuterol and Prednisone prescriptions
  - Give Albuterol every 20 minutes and reassess
  - Give Magnesium Sulfate
  - Admit to the hospital for continued management and assessment
  - Give another Albuterol/Ipratropium combo nebulizer
(15) Case 5: you are a resident on the inpatient unit and a 10-year-old patient was just sent up from the emergency room on Albuterol every two hours. Patient received one dose of prednisone by mouth in the emergency room. On initial hospital floor assessment the patient is 90 minutes after getting prior Albuterol treatment and has a respiratory rate of 28, has subcostal, suprasternal and supraclavicular retractions with inspiratory and expiratory wheezes. When asked to count to 10 in one breath she is able to count to 5. What is your next step in management?
  (Select one answer)
  - Start Albuterol every 20 minutes for the next hour
  - Give Magnesium Sulfate
  - Continue Albuterol every 2 hours
  - Give Albuterol every hour and reassess
  - Increase the dose of Albuterol and continue to give every 2 hours
(16) Case 6: you are the night team covering an inpatient unit and are making your initial rounds. On assessing a 3 year old with asthma who is on Albuterol every 3 hours right before his next treatment you note a respiratory rate of 31, end expiratory wheezes, and no retractions. Parents are at bedside and note a persistently decreased appetite and cough after playing. What is your next step in management?
  (Select one answer)
  - Advance to Albuterol every 4 hours
  - Continue Albuterol every 3 hours
  - Continue Albuterol every 3 hours AND give a dose of Magnesium Sulfate
  - Escalate to Albuterol every 2 hours AND give a dose of Magnesium Sulfate
  - Escalate to Albuterol every 2 hours
(17) Case 7: you are on the adolescent unit and seeing a 16-year-old boy on rounds who was admitted to the floor one day ago for an asthma exacerbation. Early this morning the night team advanced him to Albuterol every 4 hours and you are assessing him right before his treatment of Albuterol 4 hours after being advanced. The patient has an oxygen saturation of 93%, a respiratory rate of 22, inspiratory and expiratory wheezes, no retractions, and is able to count to 7 in one breath when asked to count to 10. He asks you if he can go home, what is your next step in management?
  (Select one answer)
  - Escalate care to Albuterol every 3 hours
  - Escalate to Albuterol every 3 hours AND give a dose of Magnesium Sulfate
  - Increase dose of Albuterol and continue every 4 hours
  - One more treatment of Albuterol every 4 hours, re-evaluate and then discharge to home after, if his exam is improving
  - Discharge home now if discharge planning is completed.
(18) Case 8: a 60 kg 15-year-old male just arrived on the floor, with the only sign out information stating he was being admitted for asthma exacerbation. You decide to start him on Albuterol every 2 hours after initially assessing him and finding he has end expiratory wheezing, subcostal retractions, and has had decreased appetite with respiratory rate of 34. What Albuterol dose will you start him on?
  (Select one answer)
  - 2 puffs MDI
  - 4 puffs MDI
  - 8 puffs MDI
  - 2.5 mg via nebulizer

*Practice*

(19) For all inpatients admitted for asthma management, how often do you use an electronic asthma order set for admission orders?
  - Never
  - Rarely
  - Sometimes
  - Often
  - Always
(20) For all inpatients admitted for asthma management, how often do you go over a written formal asthma action plan prior to discharge?
  - Never
  - Rarely
  - Sometimes
  - Often
  - Always
(21) For all inpatients admitted for asthma management, how often do you provide asthma education using patient and family education videos prior to discharge?
  - Never
  - Rarely
  - Sometimes
  - Often
  - Always
(22) How often are you able to personally assess an inpatient admitted for an asthma management before their next scheduled Q2 hour Albuterol treatment?
  - 5 times a 12-hour shift
  - 4 times a 12-hour shift
  - 3 times a 12-hour shift
  - 2 times a 12-hour shift
  - Once a shift (7 am–7 pm)

### B. Postintervention Survey

*Please Enter Your 4-Character ID*. Remember to save this ID for follow up surveys – We suggest entering into your phone or personal email under Asthma Pathway Study.

*Demographics and Background*

1. Have you attended an introductory training session on the CHAM Asthma Management Pathway
  - Yes
  - No
2. If you answered No to Question (1), answer question (2). If you answered Yes to Question (1) skip to question (3)
3. Have you completed the attestation for your independent review of a teaching presentation describing the pathway implementation?
  - Yes
  - No
4. How often do you assign a respiratory score using the CHAM respiratory score tool to a pediatric or adolescent patient admitted for asthma management?
  - Never
  - Rarely
  - Sometimes
  - Often
  - Always
5. How often do you use the CHAM asthma management pathway to make management decisions for a child or adolescent admitted for asthma management?
  - Never
  - Rarely
  - Sometimes
  - Often
  - Always
6. What year are you currently in your training?
  - Resident - Post-Graduate Year 1
  - Resident - Post-Graduate Year 2
  - Resident - Post-Graduate Year 3
  - Medical Student 4th year – matched into CHAM Pediatrics Residency for the 2014-2015 Academic year
7. How many 4-week inpatient rotations have you had at CHAM?
  - 0
  - 1-2
  - 3-4
  - 5-6
  - >7
8. How many rotations of at least 2 weeks duration at CHAM have you had in the Pediatric Emergency Room?
  - 0
  - 1
  - 2
  - 3
  - 4 or more

*Attitudes on Asthma Education. *How much do you agree or disagree with the following statements:

(8) I believe that clinical pathways or protocols that outline management hinder resident and student autonomy
  - Strongly disagree
  - Disagree
  - Neither agree nor disagree
  - Agree
  - Strongly agree
(9) I believe that clinical pathways or protocols that outline management hinder resident and student education
  - Strongly disagree
  - Disagree
  - Neither agree nor disagree
  - Agree
  - Strongly agree
(10) Clinical pathways or protocols enhance resident or student's likelihood to practice evidence-based medicine
  - Strongly disagree
  - Disagree
  - Neither agree nor disagree
  - Agree
  - Strongly agree
(11) I feel well prepared to manage an acute asthma exacerbation in emergency room and inpatient settings* without supervision*.
  - Strongly disagree
  - Disagree
  - Neither agree nor disagree
  - Agree
  - Strongly agree
(12) I believe that the implementation of a CHAM asthma pathway has had a positive effect on my education and practice skills on asthma management
  - Strongly disagree
  - Disagree
  - Neither agree nor disagree
  - Agree
  - Strongly agree

*Practice*

(13) Since the implementation of the asthma pathway, for all inpatients admitted for asthma management, how often do you use an electronic asthma order set for admission orders?
  - Never
  - Rarely
  - Sometimes
  - Often
  - Always
(14) Since the implementation of the asthma pathway, for all inpatients admitted for asthma management, how often do you go over a written formal asthma action plan prior to discharge?
  - Never
  - Rarely
  - Sometimes
  - Often
  - Always
(15) Since the implementation of the asthma pathway, for all inpatients admitted for asthma management, how often do you provide asthma education using patient and family education videos prior to discharge?
  - Never
  - Rarely
  - Sometimes
  - Often
  - Always
(16) Since the implementation of the athma pathway, about how often are you able to personally assess an inpatient admitted for asthma management before their next scheduled Q2 hour Albuterol treatment?
  - 5 times a 12-hour shift
  - 4 times a 12-hour shift
  - 3 times a 12-hour shift
  - 2 times a 12-hour shift
  - Once a shift (7 am–7 pm)
(17) How satisfied are you with the implementation of the CHAM asthma management pathway?
  - Very satisfied
  - Somewhat satisfied
  - Neutral
  - Somewhat unsatisfied
  - Very Unsatisfied
(18) If you selected (D) or (E) above please share any comments you may have describing why you are unsatisfied with the pathway: ———
(19) What are some barriers to using the CHAM asthma pathway? Check all that apply.
  - The pathway design is difficult to follow
  - The respiratory score is difficult to assign
  - Lack of education on asthma pathway use for residents
  - Providers forget to use the pathway
  - Lack of education on asthma pathway use for ancillary staff (Nursing, Respiratory Therapy)
  - Lack of education on asthma pathway use for attending physicians
  - Lack of access to the pathway or respiratory score tool when I need to reference it
  - Too many of my patients with asthma also have reasons to be excluded from the pathway (Ie. Congenital heart disease, sickle cell disease or tracheomalacia etc.)
  - Not enough time to use the asthma pathway
  - Providers sometimes disagree with management as prescribed by some parts of the pathway
  - Other (please specify) ———
(20) What are some benefits of using CHAM asthma pathway? Check all that apply.
  - Reduced length of stay of patients admitted with asthma
  - Resulted in timely discharge of asthmatics before 1 pm
  - Improved communication of patients' clinical status during shift changes
  - Improved communication of patients' clinical status amongst residents and nursing
  - Other (please specify) ———
(21) Which of these effects on resident and student education do you AGREE with (check all that apply)?
  - Decreased resident ability to know how to write asthma admission orders independently
  - Decreased my autonomy as a learner
  - Improved understanding and application of evidence based medicine
  - Improved skills in being able to communicate the current status of an asthmatic on rounds
  - Improved my ability to clinically assess a patient with an asthma exacerbation
  - Having a pathway to follow makes it less likely I will be able to independently manage asthma

*Knowledge*

(22) Case 1: you are in the Pediatric Emergency Room and a 3-year-old girl with a history of bronchiolitis presents to the asthma area. On initial assessment you count her respiratory rate to be 32 and note both subcostal retractions and nasal flaring. On inquiring about her activity level parents report a good appetite but increased cough after playing. On auscultation you hear expiratory wheezing through the entire expiratory phase. What is your next step in management?
  (Select one answer)
  - Give Albuterol once and reassess in 30 minutes
  - Give Albuterol and Ipratropium (combo neb) × 1 and reassess
  - Give Albuterol and Ipratropium (combo neb) × 3
  - Give Albuterol and Ipratropium (combo neb) × 3 and Magnesium sulfate
  - Discharge home with Albuterol and Prednisone prescriptions
(23) Case 2: you are in the Pediatric Emergency Room and a 15-year-old boy with a history of mild intermittent asthma presents to the asthma area. On initial assessment you count his respiratory rate to be 34, and note intercostal retractions. On auscultation he has both inspiratory and expiratory wheezing and is able to count to 7 when asked to count to ten in 1 breath. What is your next step in management?
  (Select one answer)
  - Give Albuterol once and reassess in 30 minutes
  - Give Albuterol and Ipratropium (combo neb) × 1 and reassess
  - Give Albuterol and Ipratropium (combo neb) × 3
  - Give Albuterol and Ipratropium (combo neb) × 3 and Magnesium sulfate
  - Discharge home with Albuterol and Prednisone prescriptions
(24) Case 3: you are in the Pediatric Emergency Room, it is the end of your shift and the 13-year-old girl you had seen in the asthma room just finished her third Albuterol/Ipratropium combo neb. She also received one dose of Prednisone 60 mg by mouth 1 hour ago. On reassessment you find her respiratory rate to be 23, with end expiratory wheezing, and subcostal retractions. On asking her to count to 10 in one breath she is able to complete the task. What is your next step in management?
  (Select one answer)
  - Admit to the hospital for continued asthma management
  - Give Albuterol once and reassess in 30 minutes
  - Give Magnesium Sulfate
  - Discharge home with Albuterol and Prednisone prescriptions
  - Discharge home with Albuterol and Ipratropium
(25) Case 4: you are in the Pediatric Emergency Room, it is the beginning of your shift and you just got sign out about a 5-year-old patient in the asthma room who received prednisone and Albuterol/ipratropium combo nebs 3 times, and was placed on 40% FiO2 via facemask for oxygen saturation of 87%. On your initial assessment she has an O2 saturation of 91% on 40% facemask, inspiratory and expiratory wheezes, subcostal retractions, and a respiratory rate of 31. Parents note a persistently decreased appetite but state that she has been drinking apple juice while in the ED, and had an increased cough when playing. What is your next step in management?
  (Select one answer)
  - Discharge home with Albuterol and Prednisone prescriptions
  - Give Albuterol every 20 minutes and reassess
  - Give Magnesium Sulfate
  - Admit to the hospital for continued management and assessment
  - Give another Albuterol/Ipratropium combo nebulizer
(26) Case 5: you are a resident on the inpatient unit and a 10-year-old patient was just sent up from the emergency room on Albuterol every two hours. Patient received one dose of prednisone by mouth in the emergency room. On initial hospital floor assessment the patient is 90 minutes after getting prior Albuterol treatment and has a respiratory rate of 28, has subcostal, suprasternal and supraclavicular retractions with inspiratory and expiratory wheezes. When asked to count to 10 in one breath she is able to count to 5. What is your next step in management?
  (Select one answer)
  - Start Albuterol every 20 minutes for the next hour
  - Give Magnesium Sulfate
  - Continue Albuterol every 2 hours
  - Give Albuterol every hour and reassess
  - Increase the dose of Albuterol and continue to give every 2 hours
(27) Case 6: you are the night team covering an inpatient unit and are making your initial rounds. On assessing a 3 year old with asthma who is on Albuterol every 3 hours right before his next treatment you note a respiratory rate of 31, end expiratory wheezes, and no retractions. Parents are at bedside and note a persistently decreased appetite and cough after playing. What is your next step in management?
  (Select one answer)
  - Advance to Albuterol every 4 hours
  - Continue Albuterol every 3 hours
  - Continue Albuterol every 3 hours AND give a dose of Magnesium Sulfate
  - Escalate to Albuterol every 2 hours AND give a dose of Magnesium Sulfate
  - Escalate to Albuterol every 2 hours
(28) Case 7: you are on the adolescent unit and seeing a 16-year-old boy on rounds who was admitted to the floor one day ago for an asthma exacerbation. Early this morning the night team advanced him to Albuterol every 4 hours and you are assessing him right before his treatment of Albuterol 4 hours after being advanced. The patient has an oxygen saturation of 93%, a respiratory rate of 22, inspiratory and expiratory wheezes, no retractions, and is able to count to 7 in one breath when asked to count to 10. He asks you if he can go home, what is your next step in management?
  (Select one answer)
  - Escalate care to Albuterol every 3 hours
  - Escalate to Albuterol every 3 hours AND give a dose of Magnesium Sulfate
  - Increase dose of Albuterol and continue every 4 hours
  - One more treatment of Albuterol every 4 hours, re-evaluate and then discharge to home after, if his exam is improving
  - Discharge home now if discharge planning is completed.
(29) Case 8: a 60 kg 15-year-old male just arrived on the floor, with the only sign out information stating he was being admitted for asthma exacerbation. You decide to start him on Albuterol every 2 hours after initially assessing him and finding he has end expiratory wheezing, subcostal retractions, and has had decreased appetite with respiratory rate of 34. What Albuterol dose will you start him on?
  (Select one answer)
  - 2 puffs MDI
  - 4 puffs MDI
  - 8 puffs MDI
  - 2.5 mg via nebulizer
(30) Which of the following patients can be included in the asthma pathway? (please check all that apply)
  - An 18 month old presenting with wheezing and increased work of breathing
  - A 3 year old presenting with a barking cough and strider
  - A 2 year old with wheezing refractory to Albuterol every 2 hours
  - A 15 year old with tachypnea, decreased breath sounds on the right and fever, found to have an opacity in the Right Lower Lobe on Chest X-Ray
(31) Which of the following is not an exclusion criteria from the asthma pathway?
  - Patient age 28 months
  - patient with sickle cell disease
  - patient with congenital heart disease
  - patient with tracheomalacia
  - patient with chronic lung disease
(32) While you took this survey, did you reference CHAM asthma management pathway or Respiratory score materials?
  - Yes
  - No
